# Pharmacological thiamine (Vitamin B1) as a treatment for alzheimer’s disease

**DOI:** 10.1002/alz.086468

**Published:** 2025-01-09

**Authors:** Gary E. Gibson, Sarah A Flowers, Howard H. Feldman, Jose A Luchsinger, Sheng Zhang

**Affiliations:** ^1^ Weill Cornell Medicine/Burke Med Res Inst, White Plains, NY USA; ^2^ University of Virginia, Charlottesville, VA USA; ^3^ University of California San Diego, La Jolla, CA USA; ^4^ University of California San Diego, Department of Neurosciences, La Jolla, CA USA; ^5^ Alzheimer’s Disease Cooperative Study (ADCS), University of California San Diego, La Jolla, CA USA; ^6^ Columbia University Irving Medical Center, New York, NY USA; ^7^ Cornell University, Ithaca, NY USA

## Abstract

**Background:**

Abnormal glucose metabolism in AD brains correlates with cognitive deficits. The glucose changes are consistent with brain thiamine (vitamin B1) deficiency. In animals, thiamine deficiency causes multiple AD‐like changes including memory loss, neuron loss, brain inflammation, enhanced phosphorylation of tau, exaggerated plaque formation and elevated advanced glycation end products (AGE). Increasing thiamine as much as 100 times with the thiamine prodrug benfotiamine diminishes all these changes.

These results plus an outstanding safety profile stimulated a double‐blind placebo‐controlled pilot trial for 12 months with benfotiamine in mild AD patients (MMSE >20). Blood thiamine increased >100 fold compared to placebo. The decline in ADAS cog was diminished by 43% (p<0.125). Worsening of the CDR was 77% lower (p<0.034). Considerable data suggests that the thiamine dependent transketolase regulates AGE, which are elevated in AD. In the pilot trial, the benfotiamine reduced AGE (p<0.044).

The following experiments sought to determine if other metabolites/lipids in serum can serve as biomarkers of the effects of benfotiamine.

**Method:**

Serum from a subset of patients on placebo and benfotiamine groups in the pilot trial was analyzed by LC‐MS/MS in parallel for comparative metabolome and lipidome.

**Result:**

A total of 315 unique metabolites and 417 lipids species were confidently identified and quantified. Differences to benfotiamine treatment were found in 25 metabolites, including thiamine, tyrosine, tryptophan, lysine, and 22 lipid species, especially phosphatidylcholines and triglycerides. Ten of 11 metabolites and 14 of 15 lipid species reported in previous literature to reflect AD progression changed in the opposite direction after benfotiamine treatment. Enrichment pathway analyses show that significantly altered metabolites are involved in glucose metabolism and biosynthesis of aromatic amino acids.

**Conclusion:**

Benfotiamine reverses the changes of metabolites/lipids reflecting AD progression. Our ongoing NIH funded multicenter trial includes blood biomarkers of thiamine, amyloid, tau, inflammation, neurodegeneration, AGE, and extensive cognitive testing. A larger sample size is necessary to test whether metabolic biomarkers are useful for disease diagnosis, prognosis, and monitoring therapeutic efficacy.

This research was partially funded by National Institute of Aging R01AG043679 (G.E.G), AG014930 (G.E.G, S.Z) and R01 AG076634 (GEG, HF, JAL)

